# *De novo* assembly and transcriptome analysis of Atlantic salmon macrophage/dendritic-like TO cells following type I IFN treatment and Salmonid alphavirus subtype-3 infection

**DOI:** 10.1186/s12864-015-1302-1

**Published:** 2015-02-18

**Authors:** Cheng Xu, Øystein Evensen, Hetron Mweemba Munang’andu

**Affiliations:** Faculty of Veterinary Medicine and Biosciences, Department of Basic Sciences and Aquatic Medicine, Section of Aquatic Medicine and Nutrition, Norwegian University of Life Sciences, P.O. Box 8146, Dep. NO-0033 Oslo, Norway

**Keywords:** Interferon, Salmonid alphavirus, Transcriptome, RNA-seq, *De novo* assembly

## Abstract

**Background:**

Interferons (IFN) are cytokines secreted by vertebrate cells involved in activation of signaling pathways that direct the synthesis of antiviral genes. To gain a global understanding of antiviral genes induced by type I IFNs in salmonids, we used RNA-seq to characterize the transcriptomic changes induced by type I IFN treatment and salmon alphavirus subtype 3 (SAV-3) infection in TO-cells, a macrophage/dendritic like cell-line derived from Atlantic salmon (*Salmo salar* L) head kidney leukocytes.

**Results:**

More than 23 million reads generated by RNA-seq were *de novo* assembled into 58098 unigenes used to generate a total of 3149 and 23289 differentially expressed genes (DEGs) from TO-cells exposed to type I IFN treatment and SAV-3 infection, respectively. Although the DEGs were classified into genes associated with biological processes, cellular components and molecular function based on gene ontology classification, transcriptomic changes reported here show upregulation of genes belonging to the canonical type I IFN signaling pathways together with a broad spectrum of antiviral genes that block virus replication in host cells. In addition, the transcriptome shows a profile of genes associated with apoptosis as well as genes that activate adaptive immunity. Further, our findings show that the profile of genes expressed by TO-cells is comparable to orthologous genes expressed by mammalian macrophages and dendritic cells in response to type I IFNs. Twenty DEGs randomly selected for qRT-PCR confirmed the validity of the transcriptomic changes detected by RNA-seq by showing that the genes upregulated by RNA-seq were also upregulated by qRT-PCR and that genes downregulated by RNA-seq were also downregulated by qRT-PCR.

**Conclusions:**

The *de novo* assembled transcriptome presented here provides a global description of genes induced by type I IFNs in TO-cells that could serve as a repository for future studies in fish cells. Transcriptome analysis shows that a large proportion of IFN genes expressed in this study are comparable to IFNs genes expressed in mammalia. In addition, the study shows that SAV-3 is a potent inducer of type I IFNs and that the responses it induces in TO-cells could serve as a model for studying IFN responses in salmonids.

## Background

Salmonid alphavirus (SAV) causes pancreas disease (PD) in Atlantic salmon (*Salmo salar* L) and rainbow trout (*Oncorhynchus mykiss*) characterized by necrosis of the exocrine pancreas, cardiomyopathy and skeletal myopathy [1]. It was first reported in 1984 [2] and later characterized as a member of the family Togaviridae [3]. Salmonid alphavirus has a positive sense, ssRNA genome which is approximately 12 kb [4]. The genome is a capped and polyadenylated positive strand RNA made of two open reading frames (ORFs) that encode structural and nonstructural proteins. Non-structural proteins are encoded by the 5′ end while structural proteins that form the envelope glycoproteins and the capsid are coded by the 3′ end [5]. Our previous studies have shown that type I interferon (IFN) inhibits the replication of SAV-3 in TO-cells [6] which has stimulated further interest to elucidate the transcriptomic changes induced by type I IFNs in salmonids.

Antiviral responses generated in response to viral infection are essential for the survival of the host. However, the assembly of an antiviral response starts at cellular level during which programmed intrinsic cell responses are initiated in a process termed ‘cell-autonomous immunity’ [7]. Chief among responses that remodel the cell’s transcriptome in response to infection is the IFN cytokine family. These cytokines induce pleiotropic biological effects by producing profiles of gene repertoires that serve as powerful signals for marshalling host defenses against microbial invasion in host cells. As pointed out by MacMicking [7], IFNs induce the expression of a broad spectrum of genes as part of an antimicrobial program designed to combat infection in all nucleated cells. Although named after their ability to interfere with virus replication in treated cells, IFNs have immunodulatory, cell differentiative, anti-angiogenic and anti-proliferative effects on cells [8]. In higher vertebrates, examination of recently identified IFN inducible genes (ISGs) using a systems biology approach reveal a highly diverse but integrated host defense program dedicated at protecting the interior of a vertebrate cell [7,9]. While these studies have opened new insights on the role of type I IFN responses in protecting mammalian cells against viral infections, studies in teleosts fish are still in their early stages and as such little is known on protective mechanisms of different ISGs on fish cells. Hence, to gain a global insight on type I IFN pathway related genes expressed in fish cells, we used a RNA-seq to analyze the transcriptomic changes induced by type I IFN and SAV-3 infection in TO-cells. In this study we wanted to find out whether TO-cells, a continuous cell line originating from Atlantic salmon (*Salmo salar* L) headkidney cells characterized to possess dendritic/macrophage like properties, would express the same profile of genes as those generated from mammalian phagocytic cells. By comparing the profile of ISGs generated from type I IFN treated cells with SAV-3 infected cells, we wanted to find out whether SAV-3 infection would produce the same profile of genes comparable to those produced by type I IFN treatment in TO-cells. The transcriptome presented herein shows that type I IFN induces the expression of a broad spectrum of ISGs and that SAV-3 is a potent inducer of type I IFN responses in TO-cells.

## Methods

### Cell culture, virus infection and IFN treatment

TO-cells originating from Atlantic salmon (*Salmo salar* L) head kidney leukocytes characterized to possess macrophage/dendritic-like properties [10,11], were propagated at 20°C in HMEM (Eagle’s minimal essential medium [MEM] with Hanks’ balanced salt solution [BSS]) supplemented with L-glutamine, MEM nonessential amino acids, gentamicin sulfate, and 10% FBS. The virus used to inoculate the TO-cells has previously been described [6] and characterized by sequencing to be salmonid alphavirus subtype 3 (SAV-3) (Genebank accession JQ799139). One batch of TO-cells was treated with 500 ng/ml of Atlantic salmon recombinant Type I in triplicates and another was infected with SAV-3 at MOI 1 when the cells were 80% confluent. Thereafter, both the type I IFN treated and SAV-3 infected cells were incubated at 15°C in maintenance media using HMEM growth media supplemented with 2% FBS. The mock group was only treated with maintenance media. After 48 hours when the cells were confluent, they were harvested and used for RNA extraction to test for type I IFN responses. All studies in TO-cells were carried out in triplicates. The recombinant type I IFN used in this study was made in our laboratory as previously described by Xu et al. [6].

### RNA isolation

Total RNA was isolated using the RNeasy mini Kit (Qiagen, Hilden, Germany) with on-column DNase treatment according to the manufacturer’s instructions. The concentration and the quality of RNA were analyzed using a Nanodrop ND1000 (Nanodrop Technologies, Wilmington, USA) and Agilent 2100 Bioanalyzer (Agilent Technologies, USA).

### Library construction, sequencing and data analysis for RNA-Seq

Equal quantities of total RNA from triplicates of the type I IFN treated, SAV-3 infected and mock-TO-cells were mixed to prepare the pooled RNA sample for RNA-Seq. Total RNA samples were treated with DNase I to degrade any possible DNA contamination. Then the mRNA was enriched using oligo(dT) magnetic beads. The mRNA was fragmented into short fragments (about 200 bp) by mixing with the fragmentation buffer. Thereafter, the first strand of cDNA was synthesized using random hexamer-primer. A buffer containing dNTPs, RNase H and DNA polymerase I was added to synthesize the second strand. The double strand cDNA was purified with magnetic beads. End reparation and 3′-end single nucleotide A (adenine) addition was then performed. Finally, sequencing adaptors were ligated to the fragments and the fragments were enriched by PCR amplification. During quality check (QC step), Agilent 2100 Bioanaylzer and ABI StepOnePlus Real-Time PCR System (Biorad.com) were used to qualify and quantify the sample library. Thereafter, library products were ready for RNA-sequencing using Illumina HiSeqTM 2000, BGI-Hong Kong. Clean reads were obtained after removal of adaptor sequences, and removal of reads having greater than 10% of unknown bases as well as removal of reads with low quality bases (base with quality value ≤ 5) greater than 50% in a read.

### Functional annotation and gene ontology classification

Once a library of clean reads was prepared, the reads were then used for transcriptome *de novo* assembly using the Trinity progam (http://trinityrnaseq.sourceforge.net/). Thereafter, assembled unigenes were used for annotation so that they could be classified for gene functioning by searching different protein databases. To do this, we used BlastX (version 2.2.23) alignment against four public protein databases; NCBI non-redundant (NR), Swiss-Prot, Kyoto Encyclopedia of Genes and Genomes (KEGG) and Cluster of Orthologous Groups (COG) at e-value < 0.00001. The best alignments from the four databases were used to determine the direction of the unigenes. As for conflicting results between the different databases, the priority order NR, Swissprot, KEGG and COG was used. Data obtained from BlastX was used to extract the coding regions (CDS) from unigene sequences and translate them into peptide sequences. Unigenes with no hits in BlastX were analyzed using ESTScan to predict their CDS and to decide their sequence direction. Unigenes with NR annotation were further analyzed with Blast2go (http://www.blast2go.org/) to obtain their gene ontology (GO) annotations, and were then further classified according to GO functions using the Web Gene Ontology (WEGO) annotation software.

### Identification of differentially expressed genes

The mapped read counts for each gene were normalized for RNA length and for the total read number in each lane using the reads per kilobase per million (RPKM) method, which facilitates comparison of the number of transcript levels generated between samples. The cutoff value for determining gene transcriptional activity was based on 95% confidence interval for all RPKM values for each gene. We used a rigorous algorithm to identify differentially expressed genes (DEGs) based on comparing the exposed with unexposed group to generate the DEGs. In this study, DEGs were generated based on comparing the RPKM mapped reads from type I IFN treated cells (IFN) versus mock TO-cells (TO), designated asTO-VS-IFN, while the second comparison was based on SAV-3 infected cells (SAV3) versus mock TO-cells (TO), designated as TO-VS-SAV3. Genes with a threshold of false discovery rate (FDR) <0.001 and an absolute value log_2_ratio > 1 were considered differentially expressed. All identified DEGs were mapped to GO annotations using the Blast2GO software (http://www.blast2go.org/).

### Validation of RNA-Seq data

To confirm the differential expression of genes revealed by RNA-Seq, 12 genes identified to be co-upregulated and 8 genes identified to be co-downregulated in TO-VS-IFN and TO-VS-SAV3 were randomly chosen for qRT-PCR validation.

qRT-PCR was performed by using the QuantiFast SYBR Green RT-PCR Kit (Qiagen) and the LightCycler 480 system (Roche). For each gene, 100 ng total RNA was used as a template in a mixture of specific primers (10 μM) (Table 1) and Master Mix in a final volume of 25 μl following manufacture’s instruction. The mixtures were first incubated for reverse transcription at 50°C for 10 min and subsequently for PCR initial activation at 95°C for 5 min, followed by 40 amplification cycles (10 s at 95°C and 30 s at 60°C). The specificity of the PCR products from each primer pair was confirmed by melting-curve analysis and agarose gel electrophoresis. The 2^–[delta][delta]Ct^ method was used to calculate the fold increase in gene expression relative to the control group. All quantifications were normalized to β-actin (endogenous gene).

**Table 1:** **Primer for quantitative real-time PCR**

**Primer name**	**Sequence**	**GeneBank Acc**
TLR3-F	TTTGATGAGTCTCCGCCAACTCCA	BK008646
TLR3-R	AATCTGCGAGGGACACAAAGGTCT	
LPG2-F	GTGGCAGGCAATGGGGAATG	FN396358
LPG2-R	CCTCCAGTGTAATAGCGTATCAATCC	
Viperin-F	GTGGAAGAGGCCATTCAGTTCAGT	BT047340
Viperin-R	AGTGCAGTTATACAGGCGGAA	
CCL19-F	TGGACCGCCTCATCAAGAAGTGC	BT125321
CCL19-R	ATGGGGGTGGAGGTGGTGGTGTT	
Galectin9-F	TTAACCTGCGTTTCAACTCGG	BT046997
Galectin9-R	TGGACCCCACTGTTCCTTCA	
Galectin3-binding-F	CCAGACCAACAGTGTTCACTTCAGC	BT059216
Galectin3-binding-R	ACGTGAAAGACATACCTGCCCTCAC	
STAT1-F	CGGGCCCTGTCACTGTTC	GQ325309
STAT1-R	GGCATACAGGGCTGTCTCT	
MHCI-F	ACCTGAAGAGAGCGACATGGA	HM181991
MHCI-R	CCCTTCCCACTTCATTTTGGA	
TRIM16-F	GGACCAAGATCTCCACTACAG	BT046063
TRIM16-R	CTGTGTTTGGGTCCAGTGTG	
IFIT5-F	GCTGGGAAGAAGCTTAAGCAGAT	BT046021
IFIT5-R	TCAGAGGCCTCGCCAACT	
PBEF-F	CACCAACAGGAGACTTTGTGACA	BT072670
PBEF-R	AAGCAGATCTGGACCGTATTCC	
IRF7B-F	GAGGAGTGGGCAGAGAACTA	NM_001171850
IRF7B-R	TTCTGGGAGACTGGCTGGG	
β-actin-F	CCAGTCCTGCTCACTGAGGC	AF012125
β-actin-R	GGTCTCAAACATGATCTGGGTCA	
Errfi-F	ACCTACATCCCCACCCTAAC	NM_001173711
Errfi-R	CAGAAACACACTGCCATCC	
AP1-F	TCTGTCCCAAGAACATCACC	BT045224
AP1-R	TCTGAGAGTCACAACTGCC	
Decorin-F	ACCTGGCTAAGCTGGGTCTA	NM_001173562
Decorin-R	TGTCCAGGTGAAGCTCTCTG	
CD209-F	TCTGACCCTGAAGCTGAAC	NM_001124633
CD209-R	ACACTCCCTACACTTCCTTAC	
C7-1-F	ATACCAATGCCAGCCTTCC	NM_001124618
C7-1-R	ATCCGACCAATCACAATCAC	
C1qt5-F	AGAAGGGAGAGAAGGGAGAC	NM_001140506
C1qt5-R	GCTGAAGGCTGATTTGGGAG	
Cacb2-F	AGAGCAGAGAAAGCAGAGAC	NM_001173925
Cacb2-R	TCATACTCCTCCTCTCCAAAC	
CtlrA -F	AAACGCATTTGTCAGATGGA	NM_001123579
CtlrA-R	GGAAGTTCATGGCTTGGTTT	

## Results

### Sequencing assessment

**Figure 1:**
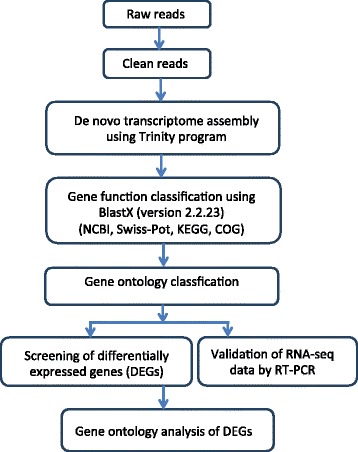
**Shows a flow chart of steps used to assemble the transcriptome from processing of raw reads followed by** ***de novo*** **assembly of the transcriptome up to gene ontology analysis of the differentially expressed genes (DEGs).**

**Figure 2:**
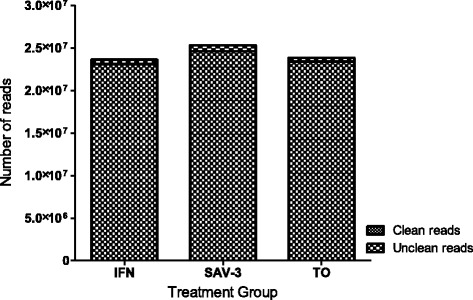
**Classification of clean reads for the IFN, SAV-3 and TO libraries.** Note that the total numbers of clean reads was >23 million reads for all the three groups.

We constructed three libraries comprising of the type I IFN treatment (IFN), SAV3 infection and mock group of TO-cells only (TO) from RNA-seq data that yielded a total of 23,113,874, 24,608,338 and 23,421,631 clean reads, respectively. A summary of the number of reads and the composition of reads generated from the RNA-seq data is shown in Table 2 while steps taken to assemble the transcriptome from clean reads are shown in Figure 1. After filtration, the percentage of clean reads in each library ranged from 97.22% to 98.23% (Figure 2). Of the total reads, genes that match to unique reads accounted for 71.31%, 78.48% and 69.27% for the IFN, SAV3 and TO groups (Table 2), respectively.

**Table 2:** **Summary of read numbers and composition of raw reads based on RNA-seq data**

**Parameters**	**rIFN treated TO-cells**	**SAV-3 infected TO-cells**	**TO-cells**
**A: Summary of reads based on RNA-seq**			
Total Reads	23,113,874 (100.00%)	24,608,338 (100.00%)	23,421,631 (100.00%)
Total Mapped Reads	18,025,140 (77.98%)	20,547,388 (83.50%)	17,901,910 (76.43%)
Unique Match	16,482,782 (71.31%)	19,313,673 (78.48%)	16,225,274 (69.27%)
Multi-position Match	1,542,358 (6.67%)	1,233,715 (5.01%)	1,676,636 (7.16%)
Total Unmapped Reads	5,088,734 (22.02%)	4,060,950 (16.50%)	5,519,721 (23.57%)
**B: Composition of raw reads**			
Reads containing adaptors	50,0443 (2.12%)	670,840 (2.65%)	393,459 (1,65%)
Reading containing N	0,0 (0.05)	0,0 (0,0)	0,0 (0,0%)
Low quality reads	22858 (0.10%)	31,578 (0,12%)	29,011 (0,12%)

### Identification of differentially expressed genes

**Figure 3:**
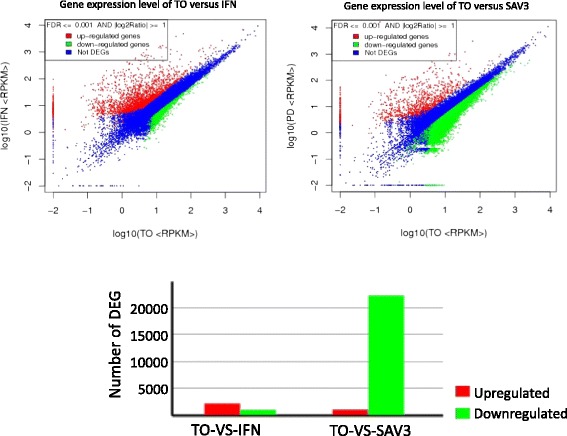
**Shows changes in the levels of differentially expressed genes (DEGs) for the TO-VS-IFN and TO-VS-SAV3.** Only DEGs with a threshold of a false discovery rate (FDR) <0.001 and absolute value log2ratio > 1 were considered differentially expressed.

Using the Trinity program, we detected a total of 58,098 unigenes from the clean reads established above. Thereafter, unigenes were screened using the criteria FDR ≤0.001 and |log2Ratio| ≥1 to identify the DEGs. Based on this approach a total of 3,149 and 23,289 DEGs were identified from the comparisons of TO-VS-IFN and TO-VS-SAV3, respectively (Figure 3). For the TO-VS-IFN, 2156 DEGs were upregulated (3.7%) and 993 DEGs were downregulated (1.7%) whereas the TO-VS-SAV3 produced 1030 up-regulated DEGs (1.8%) and 22,259 down-regulated DEGs (38.3%).

### Gene ontology analysis of DEGs

**Figure 4:**
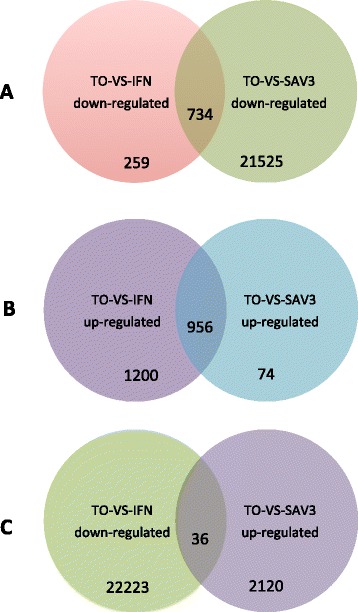
**Venn diagrams showing differentially expressed genes in the co-upregulated (A) and co-downregulated (B) group while (C) shows the differentially regulated genes in the TO-VS-IFN upregulated vs TO-VS-SAV3 downregulated group.**

**Figure 5:**
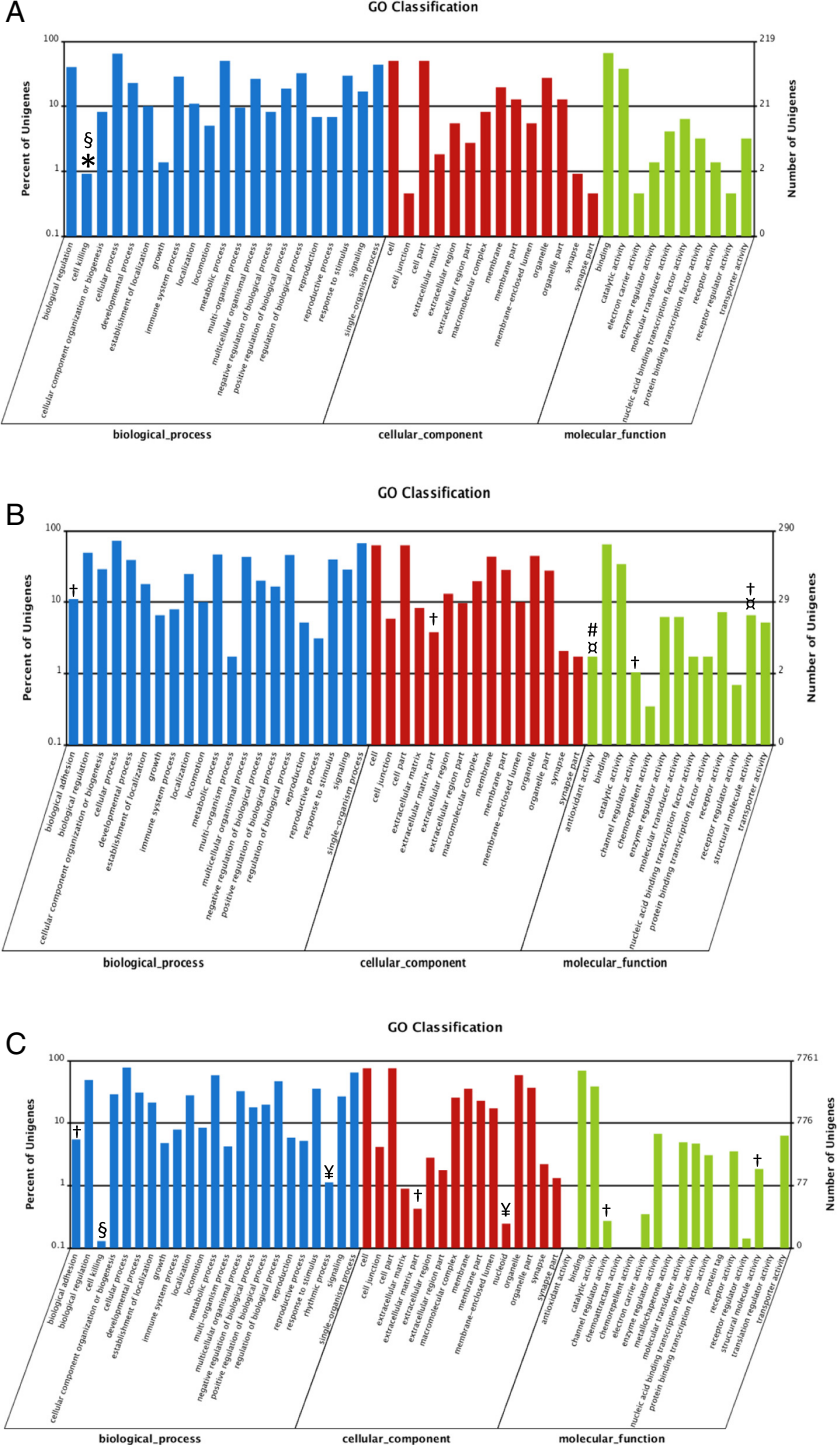
**Gene Ontology (GO) classification of co-upregulated unigenes (A) and co-downregulated unigenes (B) for the TO-VS-IFN and TO-VS-SAV3 while (C) shows downregulated unigenes only in the TO-VS-SAV3 (but not TO-VS-IFN downregulated unigenes).**

Venn diagram analysis identified 956 co-upregulated (Figure 4B) and 734 co-downregulated (Figure 4A) DEGs in TO-VS-IFN and TO-VS-SAV3 while 36 DEGs were downregulated in the TO-VS-SAV3 and upregulated in the TO-VS-IFN (Figure 4C). Based on gene ontology (GO) classification, 21, 14 and 10 functional groups were identified and classified as biological process, cellular component and molecular function, for the co-upregulated DEGs (Figure 5A), respectively. Similarly, 21, 15 and 13 functional groups were identified and classified as biological processes, cellular component and molecular function for the co-downregulated DEGs, respectively (Figure 5B). Generally, unigenes linked to biological regulation, cellular process, response to stimuli, signaling, single organism processes, cell and cell part, binding and catalytic activity were highly expressed both in the co-upregulated (Figure 5A) and co-downregulated groups (Figure 5B). However, the major difference between the co-upregulated and co-downregulated groups is that cell-killing unigenes classified under biological processes were only expressed in the co-upregulated and not the co-downregulated unigenes while antioxidant and structural molecule activity unigenes classified under molecular functions were only expressed in the co-downregulated and not the co-upregulated DEGs (Figure 5A and B). Further, Figure 5C shows GO classification of genes down-regulated in response to SAV-3 (TO-VS-SAV3) only in which 23, 16 and 12 functional groups were differentially expressed. The major differences between the three groups (Figure 5A, B and C) are that the biological adhesion, extracellular matrix part, channel regulator activity and structural molecule activity functional groups were only differentially expressed in the co-downregulated unigenes (Figure 5B) and the “TO-VS-SAV3 downregulated unigenes only” (Figure 5C) while the nucleoid and rhythmic process functional groups were only differentially expressed in the group downregulated by SAV-3 infection (Figure 5C). The cell killing functional group was higher in the co-upregulated unigenes (Figure 5A), less expressed in the group downregulated unigenes by SAV-3 infection only (Figure 5C) and absent in the co-downregulated unigenes (Figure 5B).

The X-axis shows the classification of unigenes in GO terms while the Y-axis shows the number of matched unigenes. Both the co-upregulated and co-downregulated unigenes were classified into biological process (blue), cellular components (red) and molecular function (green) (A and B). For the co-upregulated unigenes (A), biological process, cellular components and molecular functions accounted for 21, 14 and 10 functional groups while for the co-downregulated unigenes (B), biological process, cellular components and molecular functions accounted for 21, 15 and 13 functional groups. Comparing the co-upregulated (A) and the co-downregulated (B) unigenes shows that the cell-killing unigenes classified under biological process (blue) shown in asterisk (*) were only expressed in the co-upregulated group (A) while antioxidants and structural molecule activity unigenes classified under molecule function (green) (marked as ¤) were only expressed in the co-downregulated group. C shows GO classification of unigenes that were only downregulated in response to SAV-3 infection only of which the biological process, cellular components and molecular functions accounted for 23, 16 and 18 functional groups, respectively. Although the molecular function group showed 18 functional groups only 12 were differentially expressed (C). The major differences among the three groups (A, B and C) are that biological adhesion, extracellular matrix part, channel regulator activity and structural molecule activity functional groups (marked as †) were only differentially expressed in the co-downregulated unigenes (B) and the TO-VS-SAV3 downregulated unigenes only (C) while the nucleoid and rhythmic process functions (marked as ¥) were only expressed in the group downregulated by SAV-3 infection (C). The cell killing (marked as §) functional group was less expressed in C, absent in B and higher in A and finally the antioxidant activity (marked as #) functional group was only expressed in B.

### RNA-seq transcriptome profiling of type I IFN and SAV-3 induced genes

After identifying the 956 co-upregulated (Figure 4B) and 734 co-downregulated genes (Figure 4A) in the TO-VS-IFN and TO-VS-SAV3 together with the differentially regulated genes (36 DEGs) of the TO-VS-IFN versus TO-VS-SAV3 shown in Figure 4C, we manually narrowed the scope of DEGs to 60 genes induced by type I IFNs in both the TO-VS-IFN and TO-VS-SAV3 groups in order to gain a comprehensive understanding of the transcriptomic changes induced by type I IFN treatment and SAV-3 infection in TO-cells from the 60 selected genes obtained from the entire transcriptome. And as such, we identified 16 genes associated with type I IFN signaling pathway (Table 3), 22 genes belonging to different antiviral effector families (Table 4), 12 genes associated with adaptive immune responses (Table 5) and 10 apoptosis associated genes (Table 6) as shown below.

**Table 3:** **Repertoire of genes associated with type I IFN signaling pathway**

**Family**	**Gene name**	**Abbr**	**Gene ID**	**Accession no**	**Fold increase**	
					**(TO-VS-IFN)**	**(TO-VS-SAV3)**
**Co-upregulated**						
TLR	Toll-like receptor 3	TLR3	Unigene9113	DAA64469	12,47	6,12
	TLR8-like precursor	TLR8	Unigene2363	NP_001155165	38,85	16,52
NLR	NOD-like receptor 5	NLR5	Unigene34723	NP_001186995	23,13	20,58
RLR	Retinoic acid-inducible gene-I	RIG-I	Unigene7848	NP_001157171	40,77	15,32
	Melanoma differentiation associated gene 5	MDA5	Unigene6940	NP_001182108	8,41	4,87
	Laboratory of genetics and physiology 2	LGP2	CL8555.Contig1	NP_001133649	82,15	45,66
	Interferon promoter stimulating protein 1	IPS-1	Unigene12389	NP_001161824	6,07	2,95
IRF	Interferon regulatory factor 1	IRF1	Unigene15548	NP_001239293	9,68	5,04
	Interferon regulatory factor 2	IRF2	CL1885.Contig1	NP_001239280	4,00	2,97
	Interferon regulatory factor 3	IRF3	Unigene4271	ACL68544	22,38	10,30
	Interferon regulatory factor 7B	IRF7B	Unigene10251	NP_001165321	17,97	9,17
JAK	Tyrosine-protein kinase JAK1	JAK	CL2001.Contig7	NP_571148	5,96	2,26
STAT	Signal transducer and activator of transcription 1	STAT1	CL8436.Contig1	ACT79987	8,20	3,23
	Signal transducer and activator of transcription 2	STAT2	Unigene6362	NP_001138896	6,26	3,67
SOCS	Suppressor of cytokine signaling 1	SOCS1	Unigene8629	CCC15083	114,85	116,07
	Suppressor of cytokine signaling 3	SOCS3	Unigene8797	NP_998469	6,55	3,09

**Table 4:** **Repertoire of type I IFN and SAV-3 induced antiviral genes**

**Family**	**Gene name**	**ABBR**	**Gene ID**	**Accession no**	**Fold increase**	
					**TO-VS-IFN**	**TO-VS-SAV3**
**Co-upregulated**						
TRIM	Tripartite motif-containing protein 16	TRIM16	CL256.Contig2	ACI34046	14,62	5,31
	Tripartite motif-containing protein 21 (RO52)	TRIM21	CL8308.Contig1	NP_001134045	19,38	8,31
	Tripartite motif-containing protein 25	TRIM25	CL9926.Contig3	ACN11344	9,30	3,73
	Tripartite motif-containing protein 39	TRIM39	Unigene7574	XP_003977898	288,01	105,42
IFIT	IFN-induced protein with tetratricopeptide repeats 1	IFIT1	Unigene6661	AAP42146	438,18	119,60
	IFN-induced protein with tetratricopeptide repeats 5	IFIT5	Unigene6169	ACI34283	76,11	52,63
IFI	IFN alpha-inducible protein 27	IFI27	Unigene6684	XP_004082421	9,22	5,00
	IFN-induced protein 44	IFI44	Unigene8623	NP_001133872	25,10	14,65
	IFN-inducible protein Gig2-like	IFIGig2	CL7586.Contig1	ACH85338	73,07	28,29
GTPase	IFN-induced very large GTPase 1-like	VLIG-1	CL1484.Contig1	XP_003459832	917,18	421,46
	IFN-induced GTP-binding protein Mx	Mx	Unigene6767	NP_001133390	147,00	56,84
PKR	dsRNA-activated protein kinase R	PKR	CL5598.Contig1	ABU24344	9,36	3,39
ISG15	ISG15-like protein	ISG-15	CL2919.Contig1	NM_001123640	78,80	51,16
Viperin	Viperin	Viperin	CL833.Contig1	NP_001134411	155,95	91,15
Others	Megalocytivirus-induced protein 1	CsMig1	CL2853.Contig4	AFR33114	18,38	13,20
	VIG-2 protein	VIG-2	Unigene6370	NP_001117757	43,91	19,60
	Macrophage inflammatory protein 2 precursor	MIP-2	Unigene2684	ACO13449	12,80	37,38
	Lectin galactoside-binding soluble 3-binding protein	LGALS3BP	Unigene6855	NP_001135263	36,76	12,21
**Co-downregulated**						
EBPD	Enhancer binding protein delta	EBPD	CL3161.Contig1	ACF94990	−2,77	−5,82
C7-1	Complement protein component C7-1 precursor	C7-1	Unigene15784	NP_001118090	−2,09	−2,18
CXC	CXC chemokine d1	CXCd1	Unigene7278	ABA86669	−2,70	−3,33
CLEC4E	C-type lectin domain family 4 member E	CLEC4E	CL6250.Contig1	ACI67923	−6,32	−2,22

**Table 5:** **Adaptive immune genes expressed in response to type I IFN treatment and SAV-3 infection in TO-cells**

**Family**	**Gene name**	**Accession no**	**Gene ID**	**Fold increase (TO-VS-IFN)**	**Fold increase (TO-VS-SAV3)**
**Co-upregulated**					
MHC	MHC class I a	AB162342	Unigene9065	5,56	2,95
	MHC class I b	AB162343	Unigene11510	12,67	6,30
Chemokines	C-X-C motif chemokine 10 precursor	ACI69209	Unigene8163	26,09	183,97
	C-C motif chemokine 19 precursor	ACI67502	CL6894.Contig1	56,60	26,18
	C-X-C chemokine receptor type 3	NP_001133965	Unigene14449	9,19	25,20
Cytokines	IL-2 receptor	NP_001134020	Unigene16712	943,36	256,15
	Interleukin-10 receptor beta chain precursor	ACI67546	Unigene6317	25,12	13,71
Humoral	IgH locus A	GU129139	Unigene6854	6,40	2,54
	IgH locus B	GU129140	Unigene10943	14,40	6,83
	pre-B-cell colony-enhancing factor-like	NP_997833	Unigene6530	75,82	36,65
**Co-downregulated**					
Cytokines	Interleukin-12 receptor subunit beta-2-like	XP_003452899	CL7886.Contig1	−2,28	−3,75
	Perforin-1-like	XP_003446177	Unigene21875	−2,17	−2,23

**Table 6:** **Apoptosis associated genes expressed in response to type I IFN treatment and SAV-3 infection in TO-cells**

**Gene name**	**Gene ID**	**Accession no**	**Fold increase (TO-VS-IFN)**	**Fold increase (TO-VS-SAV3)**
**Co-upregulated**				
Caspase-1 precursor	Unigene6881	ACI68032	5,17	3,01
Caspase-8-like	Unigene9757	XP_001335163	5,60	2,37
XIAP-associated factor 1	CL3105.Contig1	NP_001134926	2421,31	1093,93
Galectin-9	CL1161.Contig8	ACI66798	211,64	72,25
Apoptosis-associated speck-like protein	CL4101.Contig1	XP_003197975	8,37	9,03
Tripartite motif-containing protein 16	CL8518.Contig3	ACI34059	53,16	20,02
Apoptosis regulator BAX	Unigene31520	ACI68449	27,07	10,92
CASP8 and FADD-like apoptosis regulator	Unigene16516	NP_001254595	6,07	3,69
**Co-downregulated**				
Programmed cell death 4a	CL4790.Contig1	NP_998153	−2,00	−2,54
Death-associated protein kinase 2	CL11158.Contig1	XP_683154	−2,17	−3,02

#### Genes of type I IFN signaling pathway

The repertoire of pathogen recognition receptors (PRRs), signal transducers and regulatory factors expressed in response to type I IFN treatment and SAV-3 infection in TO-cells is shown in Table 3. Generally, the fold increase was twofold higher for the type I IFN induced genes (TO-VS-IFN) than the SAV-3 induced genes (TO-VS-SAV3). Among the toll-like receptors (TLRs) only TLR3 and TLR8 were upregulated while for the RIG-like receptors (RLRs) the fold increase of LGP2 was higher than RIG-I and MDA5. Four IFN regulatory factors (IRFs) were up regulated of which the fold increase of IRF3 and IRF7 was higher than the fold increase of IRF1 and IRF2. Up-regulation of genes associated with signal transduction included tyrosine-protein kinase JAK1, STAT1 and STAT2 while the negative regulatory factors for IFN signaling upregulated included SOCS1 and SOCS3. In addition, the IFN promoter stimulating protein 1 (IPS-1) that function as an adaptor for the downstream signaling of MDA5 and RIG-I induced responses was also upregulated.

#### Repertoire of antiviral effector genes

The repertoire of antiviral genes upregulated in response to type I IFN treatment (TO-VS-IFN) and SAV-3 infection (TO-VS-SAV3) is shown in Table 4. The fold increase in genes expressed in response to type I IFN was generally twofold higher than the fold increase for genes expressed in response to SAV-3 infection. In the TRIM family, four genes were upregulated namely TRIM 16, 21, 25 and 39 while in the IFIT family only IFIT1 and IFIT5 were upregulated. Among the GTPases only Mx and VLIG-1 were detected and the fold increase of VLIG-1 was highest among all antiviral genes upregulated in response to both type I IFN treatment and SAV-3 infection in TO-cells. For the IFI family, only IFI27 and IFI44 were upregulated with the fold increase of IFI44 being higher than IFI27. Other families of antiviral genes co-upregulated included PKR, viperin, ISG15, vig-2, CsMig1 and MIP-2. Only four antiviral genes were co-downregulated by type I IFN treatment and SAV-3 infection in TO-cells and these include EBPD, CXCd1, CLEAC4E and complement protein component C7-1 precursor.

#### Adaptive immune genes

The profile of co-upregulated genes associated with adaptive immune responses is shown in Table 5. Generally, the fold increase of genes expressed in response to type I IFN treatment (TO-VS-IFN) was higher than the fold increase for genes expressed in response to SAV-3 (TO-VS-SAV3) infection except for the chemokines CXC10 and CXCR3. IL-2 receptor had the highest fold increase both for the type I IFN treated and SAV-3 infected cells. As shown in Table 5, co-upregulated genes associated with adaptive immune responses were grouped into genes associated with activation of cytotoxic T-lymphocytes (MHC-1a and 1b), chemokines (CXC10, CCL19, CXCR3), cytokine receptors (IL-2 receptor subunit beta precursor and IL-10 receptor-β-chain precursor) and humoral immune response genes (IgH locus A and B). On the other hand, co-downregulated genes included IL-12 receptor subunit β-2-like and perforin-1-like genes.

#### Repertoire of apoptosis associated genes

The repertoire of co-upregulated and co-downregulated genes associated with apoptosis is shown in Table 6. Similar to observations made for DEGs associated with the IFN signaling pathways, antiviral effectors and adaptive immunity, fold increases of genes expressed in response to type I IFN treatment (TO-VS-IFN) were higher than fold increases for genes expressed in response to SAV-3 infection (TO-VS-SAV3) except for the apoptosis associated speck-like protein. Among the co-upregulated genes, XIAF-1 had the highest fold increase, followed by Galectin-9 and TRIM-16 (Table 6). Other upregulated genes include CASP8 and FADD-like apoptosis regulator, caspase-8-like, caspase-1 precursor and apoptosis regulator BAX. Down-regulated genes include programmed cell death 4a (SAV3-4a) and death-associated protein kinase 2 (DAPK2).

### qRT-PCR validation

**Figure 6:**
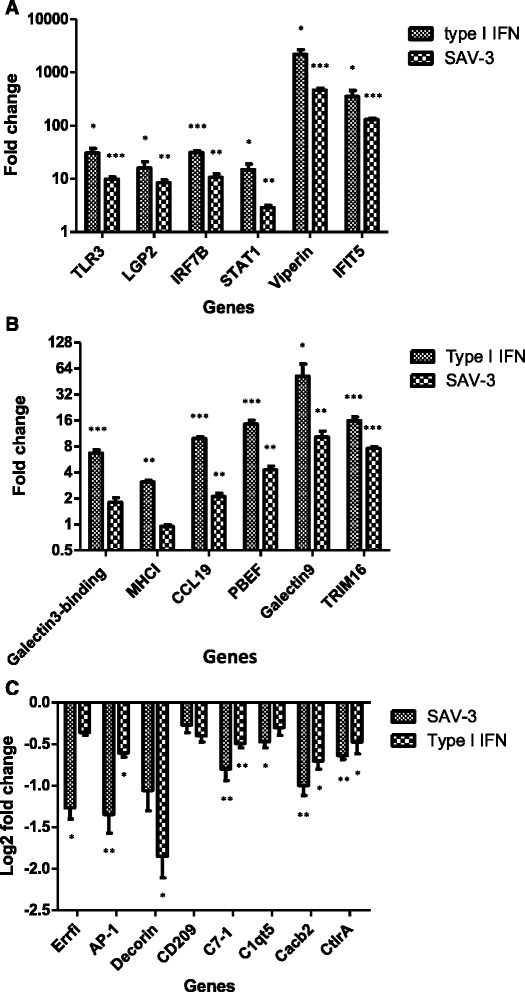
**Relative expression levels after type I IFN treatment and SAV-3 infection in TO-cells determined by qRT-PCR analysis. A** and **B** shows 12 DEGs upregulated following type I IFN treatment and SAV-3 infection of TO-cells. Data are expressed as mean fold changes of gene expression for type I IFN treated and SAV-3 infected TO-cells (n = 3) relative to the TO-cell controls (n = 3) after normalization to β-actin. SEM is presented as error bars. An asterisk (*) denotes significant upregulation (p < 0.05) when compared to the controls determined by Student’s t-test. **C** shows downregulation of eight DEGs downregulated following type I IFN treatment and SAV-3 infection. Data are expressed as log2 fold changes of gene expression for type I IFN treatment and SAV-3 infected cells (n = 3) relative to the TO-cell controls (n = 3) after normalization with β-actin.

Figure 6A and B shows relative expression of upregulated genes derived from RNA-seq data representative of the IFN signaling pathway (TLR3, LGP2, IRF7B and STAT1), antiviral genes (viperin and IFIT5), apoptosis associated genes (gelactin-9, TRIM16 and galectin-3-binding) and adaptive immune response genes (CCL19, PBEF and MHC-I) analyzed by qRT-PCR. Consistent with RNA-seq data (Tables 1, 2, 3, 4 and 5), genes generated from type I IFN treatment were higher than genes expressed from SAV-3 infection although the fold increase was not similar to observations made from RNA-seq data (Figure 6A and B). Figure 6C shows DEGs co-downregulated by type I IFN and SAV3 by qRT-PCR that were also downregulated by RNA-seq. In summary, data in Figure 6 confirms the validity of data generated by RNA-Seq by showing that the genes upregulated by qRT-PCR were also found up regulated by RNA-seq and that genes that were downregulated by RNA-seq data were also found downregulated by qRT-PCR.

## Discussion

### *De novo* assembly and transcriptome analysis

RNA-seq has emerged to be a valuable tool for studying transcriptomic changes that occur in response to microbial invasion in host cells although studies using RNA-seq on fish are limited. To our knowledge this is the first report on the use of RNA-seq to study IFN responses in fish cells. Although several computer based *de novo* assembly tools (e.g. Trans-AbySS, Oasis, SOAP2denovo and Trinity) have been developed, only the trinity program was used for sequence assembly in this study given that several studies have shown that Trinity is a powerful tool that has proved to be useful for annotating transcriptomes for different vertebrate species across taxa [12,13]. Compared to other *de novo* transcriptome assemblers, Trinity recovers more full length transcripts from RNA-seq data without a reference genome with a sensitivity similar to methods that rely on genome alignments [12] and as such Trinity was considered to be a better tool for use in TO-cells given the limited number of annotated genes that would serve as reference genes in salmonids. Using Trinity, this study demonstrates that short reads from illumina RNA-seq can be assembled into protein sequences used to identify DEGs in fish cells that can be matched to orthologous genes found in mammals. Therefore, from a comparative immunology standpoint, *de novo* assembly of transcriptomes can be used to compare orthologs of immune genes expressed in lower vertebrates, such as teleosts fish, with those found in higher vertebrates. For example, the DEGs generated in this study were matched to genes associated with IFN signaling pathway, antiviral effector functions, apoptosis associated genes and genes that activate adaptive immune responses that conform to IFN inducible genes expressed in higher vertebrates. And as such, given the limitation on annotated genes induced by type I IFN in fish, we used a comparative approach and compared genes expressed in this study with orthologous genes expressed in mammalian cells to gain a better understanding of the transcriptomic changes that occur in response to type I IFN treatment and SAV3-virus infection in TO-cells as discussed in detail below.

### Genes of the type I IFN signaling pathway

Recognition of pathogen associated molecular patterns (PAMPs) by PRRs constitutes the first line of defense used by host cells in sensing microbial invasion. Three PRR families were upregulated namely TLRs, RLRs and NLRs in this study. Among the TLRs, only TLR3 and TLR8 that are sensors of nucleic acids released in the endosomal compartments of macrophages and DCs [14] were upregulated, which augments the notion that TO-cells possess macrophage/dendritic cell like properties [10]. Unlike TLRs, RLRs comprising of MDA5, RIG-I and LGP2 are primary sensors of the 5′-triphosphate RNA expressed by viruses in the cytoplasm [15] and their upregulation in the case of SAV-3 infection in TO-cells supports the consensus view that they bind to the 5′-ppp-RNA expressed by alphaviruses during replication [16]. While TLRs use the MyD88/TRIF adaptor, RIG-I and MDA5 uses the IPS-1 adaptor in their downstream signaling [17], which was also upregulated in this study. Further studies in mammalia show that RLR signaling converges on pathways utilized by TLR3 and TLR8 resulting in the production of similar ISGs [17].

Type I IFNs bind to the IFNα receptor (IFNAR), which activates JAK1 and TYK2 that phosphorylates STAT1 and STAT2, which form heterodimers, and in association with IRF9, forming a trimeric complex of IFN-stimulated gene factor 3 (ISGF3) that translocates to the nucleus where it binds IFN-stimulated response elements (ISRE) in the promoters of ISGs genes to drive their expression [18]. ISGs interfere with virus replication to establish a cellular antiviral state. Of the four upregulated IRFs, expression of IRF3 and IRF7 was higher than IRF1 and IRF2 suggesting that IRF3 and IRF7 played a major role in inducing type I IFN responses in TO-cells. In additions, these findings suggest that IRF3 and IRF7 regulate the TLR3 and TLR8 pathways which is in line with observations in mammalia where it has been shown that IRF3 and IRF7 are master regulators of TLR3 and TLR8 pathways [14]. Finally, SOCS1 and SOCS3 block JAK activity and STAT recruitment to the receptor thereby creating a negative signaling that prevents excess cytokine signaling that could impair the normal homeostasis and cellular function of IFN producing cells [18,19]. Hence, the expression of SOCS1 and SOCS3 in this study could have been for regulating the negative feedback of IFN signaling induced in TO-cells. Overall, genes expressed in response to type I IFN treatment and SAV-3 infection in this study conform to genes of the canonical type I IFN signaling pathway expressed in higher vertebrate cells.

### Antiviral effector genes

Antiviral genes induced by type I IFN exert their effector functions by inhibiting the replication of virus at different stages of virus replication cycles. For examples, PKR inhibits cellular mRNA translation which in turn prevent viral protein synthesis [20] while ISG15 achieves its antiviral function by conjugating to target proteins followed by altering their function [21]. Viperin interferes with assembly and release of virus particles by disrupting the endoplasmic reticulum transport system that translocates viral particles to the plasma membrane [22]. Hence, the upregulation of these genes in this study could opt for similar functions as those reported in higher vertebrates.

The TRIM family is primarily induced by type-I IFNs expressed in macrophages, dendritic cells, lymphocytes and fibroblasts [23]. Recently, various TRIMs have been identified in different fish species [24-26] inclusive of TRIMs 16, 25 and 39 expressed in this study. TRIM proteins block virus infection using different mechanisms [7]. For example, TRIM5α restricts virus entry by binding to the capsid of HIV virus to block uncoating [27,28] while TRIM22 interferes with pre-genomic RNA synthesis and protease activity of hepatitis B virus [29]. Hence, it is likely that TRIM 16, 21, 25 and 39 expressed in TO-cells in this study have antiviral effects targeted at different stages of virus replication. Apart from their antiviral effector functions, TRIMs also participates in the induction of IFN synthesis. For example, TRIM25 is involved in the synthesis of IFN-β through the RIG-I pathway [30]. Thus, it is likely that TRIMs expressed in this study are involved in the synthesis of IFNs. However, there is need for detailed studies to elucidate the mechanisms used by these TRIMs in restricting SAV-3 replication in fish cells and to determine their role in the synthesis of type I IFNs in TO-cells.

The IFIT family encodes genes induced by IFN treatment, virus infection or PAMP recognition and they confer antiviral protection through disruption of protein-protein interactions in the host translation-initiation machinery [31]. Recent revelations show that IFITs can specifically recognize ssRNA bearing a 5′-(5′-ppp-) triphosphate group [32], thereby acting as a sensor for detecting viral ssRNAs. Single-stranded 5′-ppp-RNAs, which lack 2′-O-methylation of the 5′ cap and bear a 5′-ppp group, are specifically from viruses, which serve as a molecular signature for distinguishing self from non-self mRNAs [33,34]. Crystallography has shown that only single stranded 5′ppp-RNAs bind to IFIT1 and IFIT5 [33]. Hence, it is likely that the IFIT1 and IFIT5 expressed in this study binds to the 5′-ppp-RNA of SAV-3 to create an antiviral state against the virus in TO-cells [35].

IFN-inducible GTPases currently recognized in humans are made of four families namely the myxovirus resistant proteins (Mxs), guanylate-binding proteins (GBPs), immunity-related GTPase proteins (IRGs) and very large inducible GTPase proteins (VLIGs). Among these only Mx and VLIGs were expressed in response to type I IFN treatment and SAV-3 infection in this study. Thus far, only Mx has been widely studied in salmonids [36-38] while the antiviral effector mechanisms of VLIGs in fish cells are yet to be elucidated.

Other antiviral genes upregulated in response to type I IFN treatment and SAV-3 infection in TO-cells include IFI27, IFI44, CsMig1, MIP-2 and vig-2 protein while the down regulated genes include CXCd1 and CLEAC4E. Although some of these genes, such as vig-2, have previously been reported in fish [39] their functional mechanisms have not been established this far and there is need to elucidate the antiviral mechanisms of these genes in fish cells.

### Type-I IFN apoptosis associated genes

Apoptosis, a form of programmed cell death, is a mechanism used by multicellular organisms to get rid of unwanted cells in a systematic manner [32,40]. The central feature of apoptosis, unlike necrosis, is the containment of cellular materials in membranous structures in which apoptotic particles are phagocytized without leaking the intracellular contents to the extracellular *milieu* [41,42]. To attain this, apoptotic cells induce signaling pathways using IFN induced genes to facilitate the removal of unwanted cells from the host. Among the apoptosis associated genes expressed in this study, XAF-1 had the highest response to type I IFN treatment and SAV-3 infection followed by Gelactin-9. XIAF-1 has been shown to block the activities of XIAP which is a known inhibitor of apoptosis (IAP) in human cells [43,44] while Galectin-9 is vital for T-cell apoptosis where it participates in killing activated or infected T-cells following an immune response [45]. Other apoptosis associated genes expressed in this study include the apoptosis associated speck-like protein which contains a caspase recruitment domain (CARD) linked to induction of apoptosis signaling pathways [46] while caspase 8 belong to the family of proteases that function in the initiation and execution of cell disassembly in response to apoptosis signals [47]. Although the functional mechanisms of these genes are well studied in higher vertebrate cells, their mode of action in fish cells is yet to be elucidated.

### Adaptive immune response genes

Type I IFNs enhance antigen presentation by upregulation of MHC-I and differentiation of virus specific cytotoxic T-lymphocytes thus providing an important link between innate and adaptive immunity [48]. The repertoire of adaptive immune genes upregulated in this study conforms to genes engaged in activation of the adaptive immune response. For example, expression of MHC class Ia and Ib genes was accompanied by upregulation of chemokines essential for recruitment and activation of T-cells such as CXCR3, which is selectively expressed in activated T-cells and not in other leukocyte subpopulations [49]. CXCR3 functions as a receptor for CXCL10 which plays an important role in the development and maturation of T-cells [50]. CCL19 is strongly chemotactic for naïve CD4 and CD8-T-cell and its expression plays an important role in the homing of naïve T-cell to MHC-I molecules expressing surface antigens on DCs [51]. By executing its chemotactic role, CCL19 initiates the activation of naïve CD4 and CD8 T-cells into effector T-helper (T_H_) cells and cytotoxic T-lymphocytes (CTLs) [51], respectively.

Apart from enhancing the maturation of naïve T-cells into cytotoxic T-lymphocytes, type I IFNs also play an important role in the maturation of B-cells. In the present study this notion was supported by upregulation PBEF a cytokine that acts on early B-cell lineage precursor cells and regulates the early and late events leading to maturation of B-cell [52]. In addition, upregulation of IgH locus A and B is suggestive of reorganization of the B-cell receptors induced by type I IFNs during the early developmental stages. Further, upregulation of IL-2-receptor which regulates the activation of naïve T-helpers cells to activated T_H_2 cells [53] that in turn play a helper role in the maturation of naïve B-cells into immunoglobulin secreting cells [54] further supports the role of type I IFNs in activating the adaptive immune response [55]. Although the functional mechanisms of these genes are well studied in higher vertebrates, their upregulation in this study suggest that they could have the same functional roles in fish cells.

### Induction of type I IFN responses by SAV-3 in TO-cells

Data presented here suggests that SAV-3 is a potent inducer of type I IFNs in TO-cells. Our previous studies show that despite SAV-3 induces a strong IFN response, the virus replicates vividly in TO cells [6]. When cells are pre-treated with recombinant IFN, 4 to 24 hrs prior to infection, with recombinant IFNα TO cells are protected against virus induced cytopathic effects and virus replication is inhibited [6]. These findings are consistent with observations made for other alphaviruses in mammalia showing that pretreatment with IFN inhibits alphavirus replication of *in-vivo* [56,57]. For SAV-3 it is not understood why the virus can replicate in vitro under strong IFN responses. Studies in Chikungunya virus (CHIKV), another alphavirus, show that virus replication is resistant to IFN inhibition once replication is established [58] and it was shown that CHIKV uses the nsP2 to suppress the antiviral effect of IFN by interfering with the JAK-STAT signaling pathway enabling the virus to replicate in the presence of IFN. TO-cells used in this study were not pretreated with IFN prior to SAV-3 infection and concordant with previous findings once infection was established, IFN released through downstream signal transduction had no inhibitory effects in the subsequent replication of the virus [6]. Although we did not determine the mechanisms used by SAV-3 to circumvent the IFN antiviral effects produced by downstream signal transduction, several studies have shown that alphaviruses use different mechanisms to block the antiviral effects of IFN once infection is established [59,60]. Put together, these studies accentuate the importance of timing on the inhibitory effects of IFNs on the replication of alphaviruses in infected cells. Based on data presented here and our previous findings [6], it can be concluded that TO-cells pretreated with IFNα (4-24hrs) before infection inhibit SAV-3 replication while the IFN response induced from the SAV-3 infection is not sufficient to inhibit virus replication [6]. Thus, there is need for more detailed investigations to elucidate the mechanisms that inhibit the antiviral effects of IFN produced by downstream signal transduction after SAV-3 infection in TO-cells.

### Downregulation of host cellular gene expression by SAV-3 infection in TO-cells

It is interesting to note that TO-cells infected by SAV-3 (TO-VS-SAV3) had by far a large proportion (22,259 DEGs) of down-regulated genes compared to IFN treated cells (TO-VS-IFN) (993 DEGs). Similar observations have been made for other alphaviruses [61,62] and several other viral families [63-67] in which virus infection has been shown to downregulate the expression of several genes regulating cellular transcription during virus replication as a survival strategy leading to persistent infections. For example, Gorchakov et al. [61] have shown that replication of Sindbis virus downregulates major cellular processes such as the transcription and translation of mRNAs in infected cells. In their studies, they noted that inhibition of cellular mRNA transcription is a critical phenomenon used by viruses to suppress the expression of cellular stress inducible genes in vertebrate cells as a survival strategy to enhance their replication. Hence, this would account for the downregulation of a large proportion of genes linked to cellular, biological and molecular functions in TO-cells that were only infected by SAV-3 (TO-VS-SAV3) and not those treated with IFN (TO-VS-IFN) in this study. Similarly, in our previous studies we showed protein shutdown in TO-cells infected with SAV-3 only which was not seen in untreated TO-cells [6] which augments our current findings. Put together, these studies suggest that SAV-3 infection could be downregulating a wide range of host cellular genes to enhance its replication in TO-cells using mechanisms similar to those seen for other alphaviruses in higher vertebrates [61,62]. However, there is a need for detailed investigations to underpin the exact mechanisms used by this virus to dowgnregulate the expression of host cellular genes and to demonstrate how suppression of these genes could enhance the replication of SAV-3 in TO-cells.

## Conclusion

In this study, we present a *de novo* assembly and analysis of a transcriptome of DEGs generated in response to type I IFN treatment and SAV-3 infection in the macrophage/dendritic like TO-cells derived from Atlantic salmon headkidney leukocytes. Transcriptomic changes reported here show a profile of genes belonging to the canonical type I IFN signaling pathway together with a broad spectrum of antiviral effector genes that block virus replication at different stages of the virus replication cycle. In addition, the transcriptome also shows a profile of genes associated with apoptosis as well as a repertoire of genes associated with activation of adaptive immunity. Our findings also show that the profile of type I IFN pathway related genes expressed by TO-cells is comparable to orthologous genes expressed by mammalian macrophages and dendritic cells. Further, the study shows that SAV-3 is a potent inducer of type I IFN responses in TO-cells and that it could serve as a reliable model for studying IFN protective mechanisms in fish cells. However, it is vital to note the repertoire of type I IFN induced genes reported here only shows a profile of up- and down-regulated genes, but it does not show the exact mechanisms used by these genes to protect host cells. And as such, future studies should seek to elucidate the functional mechanisms used by these genes in protecting fish cells. In summary, this study shows a *de novo* assembly of a transcriptome of IFN induced genes in response to type I IFN treatment and SAV-3 infection in TO-cells that were matched to their orthologs in higher vertebrates.

## Data access

The RNA-sequencing data generated in this study has been deposited in the National Center for Biotechnology Information (NCBI) Gene Expression Omnibus (GEO) database accession number GSE64095 (www.ncbi.nih.gov/geo Accession number GSE64095).
